# The Unholy Trinity: Childhood Trauma, Adulthood Anxiety, and Long-Term Pain

**DOI:** 10.3390/ijerph17020414

**Published:** 2020-01-08

**Authors:** Natalia Kascakova, Jana Furstova, Jozef Hasto, Andrea Madarasova Geckova, Peter Tavel

**Affiliations:** 1 Olomouc University Social Health Institute, Palacky University Olomouc, Univerzitni 22, 77111 Olomouc, Czech Republic; jana.furstova@oushi.upol.cz (J.F.); j.hasto.tn@gmail.com (J.H.); andrea.geckova@upjs.sk (A.M.G.); peter.tavel@oushi.upol.cz (P.T.); 2 Psychiatric-Psychotherapeutic Outpatient Clinic, Heydukova 27, 81108 Bratislava, Slovakia; 3 Department of Social Work, St. Elizabeth College of Health and Social Work, Palackeho 1, 81102 Bratislava, Slovakia; 4 Department of Psychiatry, Slovak Medical University, Faculty of Medicine, Limbova 12, 833 03 Bratislava, Slovakia; 5 Department of Health Psychology, Faculty of Medicine, P. J. Safarik University, Trieda SNP 1, 04011 Kosice, Slovakia

**Keywords:** childhood trauma, adulthood anxiety, chronic pain condition, anxiety disorder, community sample, clinical sample

## Abstract

Background: Childhood trauma is considered to be a risk factor for developing anxiety as well as chronic pain. The aim of this study was to assess the association between childhood trauma and reporting anxiety and long-term pain conditions in the general and clinical populations. Methods: Respondents from a representative sample in the Czech Republic (*n* = 1800, mean age: 46.6 years, 48.7% male) and patients with a clinically diagnosed anxiety or adjustment disorder (*n* = 67, mean age: 40.5 years, 18.0% male) were asked to report anxiety, various chronic and pain-related conditions, and childhood trauma (The Childhood Trauma Questionnaire, CTQ) in a cross-sectional questionnaire-based survey conducted in 2016 and 2017. Results: Reporting emotional abuse (Odds ratio OR from 2.14 to 14.71), emotional neglect (OR from 2.42 to 10.99), or physical neglect (OR from 2.24 to 3.30) was associated with reporting anxiety and long-term pain both in the general and clinical populations and reporting physical abuse moreover with reporting anxiety or adjustment disorder with concurrent long-term pain (OR from 4.04 to 6.39). Conclusion: This study highlights the relevance of childhood trauma as a possible factor contributing to anxiety with concurrent pain conditions in adulthood in both the general and clinical populations.

## 1. Introduction

Adverse childhood experiences often bring undesirable consequences. There is evidence that experience of childhood trauma may lead to anxiety and long-term pain in adulthood [1,2,3], which may also reach the level of psychopathology [4,5,6]. Moreover, in a clinical setting many patients with anxiety symptoms report some pain condition and, vice versa, many patients with chronic pain suffer from anxiety. Research on anxiety symptoms and pain has increased rapidly in recent decades [7,8,9,10,11]. Although they have already been extensively studied, the links between childhood trauma and symptoms of anxiety and pain should be explored further to better understand the underlying common processes [12,13,14,15]. This study compares a community sample reporting anxiety and long-term pain conditions with a clinical sample of patients with anxiety or adjustment disorders reporting pain, regarding a history of childhood trauma. The control samples consist of individuals from the community reporting no chronic conditions and those reporting some chronic conditions but not anxiety or/and pain.

### 1.1. Epidemiology of Chronic Pain and Anxiety

Chronic pain is a major public health problem that negatively affects quality of life, sleep, and work and increases health care costs as well as mortality [16,17]. The prevalence of chronic pain in the general population of Europe is estimated to be 19.0% [18].

Symptoms of anxiety are a core indicator for anxiety disorders and are often associated with adjustment disorders as well [19]. Anxiety disorders are highly prevalent in the population. In a large European epidemiological study the lifetime prevalence of any anxiety disorder reached 13.6% [20]. In a recent representative cross-sectional household survey of Czech adults [21], the prevalence of current anxiety disorders was estimated at 7.3%. Adjustment disorders receive little attention in research, even though they are the most commonly used diagnosis among mental health specialists according to worldwide surveys [22,23].

Many population-based studies have highlighted the importance of the relationship between anxiety and chronic pain (review in [24]). In a Canadian representative sample [8] and in a large cross-national survey from 17 countries around the world [25] the prevalence of anxiety disorders was nearly twice as high in persons with chronic pain compared to the healthy population.

An extensive discourse has been taking place about the causality of anxiety and pain. Analyses of temporal relationship showed that anxiety disorders mostly preceded the onset of pain [13,26,27] and that individuals with mental disorders, namely depression and anxiety disorder, are at higher risk of developing subsequent back/neck pain [10]. According to a cross-sectional community survey of adults in 10 countries [13], childhood adversities and early onset of mental disorders have independent, broad-spectrum effects that increase the risk of diverse chronic physical conditions later in life. In the 10-year follow-up study [12] anxiety and mood disorders were found to partially mediate and moderate the relationship between early trauma and painful medical conditions.

On the other hand, it is clear from several longitudinal studies that the relationship between symptoms of anxiety and pain is bidirectional [28]: pain may cause feelings of anxiety, which potentiates sensitivity to pain and can contribute to pain chronification [29,30]. There is no doubt that anxiety and pain in clinical practice are strongly related, which has already been well validated by many research studies described above; and what about childhood trauma, the third item of the mentioned ‘unholy trinity’?

### 1.2. Childhood Trauma in Chronic Pain and Anxiety

Several decades ago, Engel [31] pointed out that on the basis of clinical observations psychological deprivation and traumatization appear to be more frequent in some patients with chronic pain. The results of a meta-analysis of the association of chronic pain and childhood trauma provide evidence [1] that individuals who report abusive or neglectful childhood experiences are at increased risk of experiencing chronic pain in adulthood relative to individuals not reporting abuse or neglect in childhood. A recent German population study [2] showed that emotional, physical, and sexual abuse specifically seem to have a long-lasting effect on the experience of pain in the general population.

There is evidence that childhood trauma plays a substantial role in the later occurrence of psychopathology in adolescence or adulthood, including anxiety and depression [6,32,33,34,35].

Previous research indicates that chronic pain and anxiety can share common risk factors [36]. One common risk factor may be early experience of childhood trauma, e.g., emotional abuse, physical abuse, sexual abuse, and emotional and physical neglect, which can—via changed neurohumoral regulation of the hypothalamic-pituitary-adrenal axis and effects on the autoimmune system—produce vulnerabilities for later psychopathology as well as pain conditions [37,38,39]. In previous studies, physical and/or sexual abuse were the most common forms of childhood abuse studied in association with pain [40,41]. However, there is evidence that emotional abuse and neglect are no less relevant in their association with pain [42] as well as with anxiety disorders [6,34,43] or in the concurrence of pain and anxiety [12,44,45].

To address the above-mentioned issues, we had the following aims in this study: (1) To describe the prevalence of various childhood trauma experiences in the general and clinical populations. (2) To assess whether reporting childhood trauma increases the odds of suffering from pain and anxiety in the general and clinical populations.

## 2. Methods

### 2.1. Sample

This study is based on a comparison of four samples: three samples originate from a health survey conducted on a representative population sample in the Czech Republic; the fourth sample is clinical. The same set of questions was used in all four samples.

The health survey was conducted in 2016 on a general population of the Czech Republic. A total of 2184 respondents randomly selected from a list of inhabitants of the Czech Republic, stratified by gender, age, and 14 regions, were asked to participate in a larger study on health. Of those asked to participate, 384 refused, more men and younger age groups, mostly due to a lack of time, non-confidence, and length of the questionnaire or reluctance. Ultimately, data from 1800 respondents were collected by trained administrators using face-to-face interviews during September and November 2016. The selected group of 1800 participants is a representative sample of the population of the Czech Republic over the age of 15 in relation to sex (48.7% men), age composition (age 15 to 90 years old, mean age: 46.61), and regional affiliation.

Respondents were asked if they suffer from some long-term chronic condition (e.g., hypertension, asthma, allergy, anxiety or some pain-related condition—such as migraine, back pain, arthritis, pelvic pain, or pain of unclear origin). For the purposes of this study, we identified 405 respondents reporting no chronic conditions (the ‘healthy’ community sample reporting no chronic conditions), 506 respondents reporting some ‘other chronic condition’, but not anxiety or/and pain (e.g., hypertension, allergy, etc.), and 91 respondents reporting anxiety and some pain-related condition (the community sample reporting anxiety and pain only or in addition to some other chronic condition). This means that respondents reporting anxiety and pain-related conditions may possibly suffer from some other chronic conditions.

**Figure 1:**
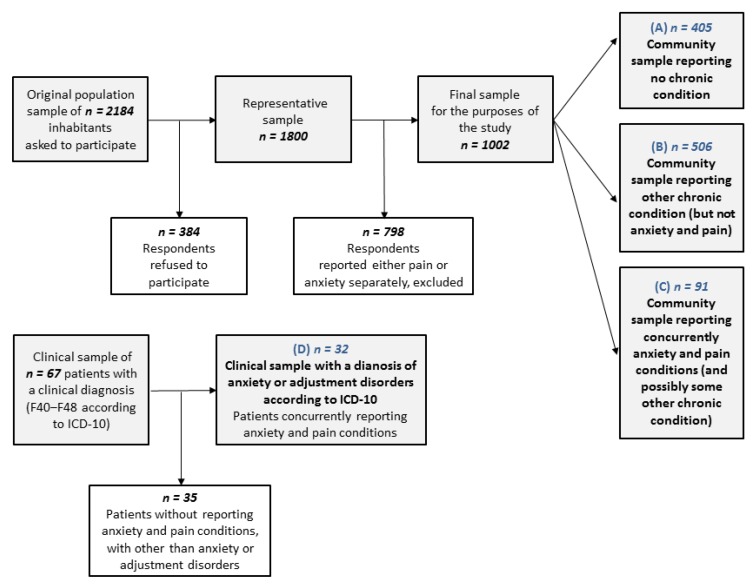
Scheme of data selection for the purpose of the study analyses.

The data from the clinical sample were collected between January and June 2017 at the Sternberk Psychiatric Inpatient Hospital (9 patients) and the Kromeriz Psychiatric Inpatient Hospital (58 patients). In total, the randomly collected clinical sample consisted of 67 patients with a diagnosed disorder from the spectrum of anxiety, adjustment and somatoform disorders (F40‒F48) according to the International Classification of Diseases (ICD-10) criteria. For the purposes of our study analyses, 32 patients (mean age: 39.1 years, 18.8% male) with a diagnosis of anxiety disorders or adjustment disorders according to ICD-10 who concurrently reported anxiety and some pain condition described above were selected from the clinical sample. Figure 1 summarizes the final population and clinical sample selection.

No data was missing in the community sample. In the clinical sample, less than 1% of data was missing. It was assumed that data are missing at random (Little’s Missing Completely At Random (MCAR) test: *χ*^2^(134) = 149.1, *p* = 0.18). Thus, in the clinical sample, we performed a multiple imputation of the missing data on the item level 20 times. The Hmisc package in the R software was used for the imputation of missing data.

Respondents agreed to participate in the study by signing an informed consent prior to the study. This study was approved on 14 June 2016 by the Ethical Scientific Committee of Palacky University Olomouc (no. 2016/3) and conducted in accordance with the protection of personal data (act. no. 101/2000 Coll.).

### 2.2. Measures

#### 2.2.1. Sociodemographic Data

Participants reported gender (male or female), age (continuous), living arrangement (living with a partner in a marriage or a partnership, alone, with parents or siblings) and education (primary, skilled operative, high school graduated, and college or university).

#### 2.2.2. Long-Term Health Complaints

Long-term health complaints were measured by the item “Do you have some long-lasting disorder or disability? Please, mark all possibilities which are related to you.” Respondents chose from following list: ischemic heart disease, hypertension, cerebral insult/hemorrhage, chronic pulmonary disease, asthma, cancer, diabetes, obesity, arthritis, back pain, gastric and duodenal ulcer, inflammatory bowel disease, dermatitis (eczema), allergy, migraine, pain of unclear origin, pelvic pain—in women, diseases of thyroid gland, anxiety, other, or no disease.

#### 2.2.3. Childhood Trauma

The Childhood Trauma Questionnaire (CTQ) is a retrospective self-report measuring the severity of five different types of childhood trauma: emotional abuse (EA), physical abuse (PA), sexual abuse (SA), emotional neglect (EN), and physical neglect (PN) [46]. Each subscale has five items rated on a five-point Likert-type scale with response options ranging from 1, ‘never true’ to 5, ‘very often true’. This tool offers the possibility of assessing the relevance of the abuse and neglect according to scores reached in each subscale, with four levels of maltreatment: none (or minimal), low (to moderate), moderate (to severe) and severe (to extreme) [47]. We used Walker’s procedure of severity ratings in the present study [48]. According to Walker’s approach, PA and PN include all cases from ‘slight to moderate’ up to ‘extreme’ childhood trauma (cut-off score 8), and SA and EN include all cases from ‘moderate to severe’ up to ‘extreme’ childhood trauma (8 for SA, 15 for EN). For EA the cut-off point is in the middle of the ‘slight to moderate’ level (cut-off score 9). The Czech version of the CTQ has been shown to be both reliable and valid [49]. In this study, Cronbach’s alpha of the CTQ scale reached values of 0.72 in the community sample and 0.88 in the clinical sample, respectively.

### 2.3. Statistical Analyses

The distribution of the raw scores of the questionnaire subscales was evaluated using histograms, and their normality was verified using the Shapiro–Wilk’s normality test. Since the data was not normally distributed, non-parametric methods were used for the statistical analyses. Differences in percentages of occurrence of individual types of trauma were assessed using a test of proportions with Bonferroni correction. To assess the odds of having pain syndromes in adulthood, depending on childhood trauma experience, multinomial logistic regression models were used. There was a four-level dependent variable: 0—being healthy (reporting no chronic condition); 1—reporting chronic conditions, but not anxiety or/and chronic pain; 2—reporting anxiety and chronic pain conditions (possibly with other chronic conditions); 3—suffering from a clinical diagnosis with reporting anxiety and pain conditions. As predictors, we used childhood trauma (CTQ subscale scores) dichotomized according to Walker’s scoring system [48], with no childhood trauma being the reference category. All regression models were adjusted for the age and gender of the respondents. All analyses were performed using the statistical software package IBM SPSS version 21 (IBM Corp., Armonk, New York, NY, USA) and R 3.6.0. (R Foundation for Statistical Computing, Vienna, Austria).

## 3. Results

### 3.1. Sociodemographic Characteristics

Four groups of respondents were studied (see Figure 1): (A) Community sample reporting no chronic condition (*n* = 405); (B) Community sample reporting other chronic conditions, but not anxiety or/and pain (*n* = 506); (C) Community sample reporting anxiety and some pain condition (*n* = 91); (D) Clinical sample (respondents with a clinical diagnosis of anxiety or adjustment disorder who concurrently reported anxiety and some pain condition, *n* = 32). The sociodemographic characteristics of these groups are presented in Table 1.

### 3.2. Prevalence of Various Childhood Trauma Experiences

**Figure 2:**
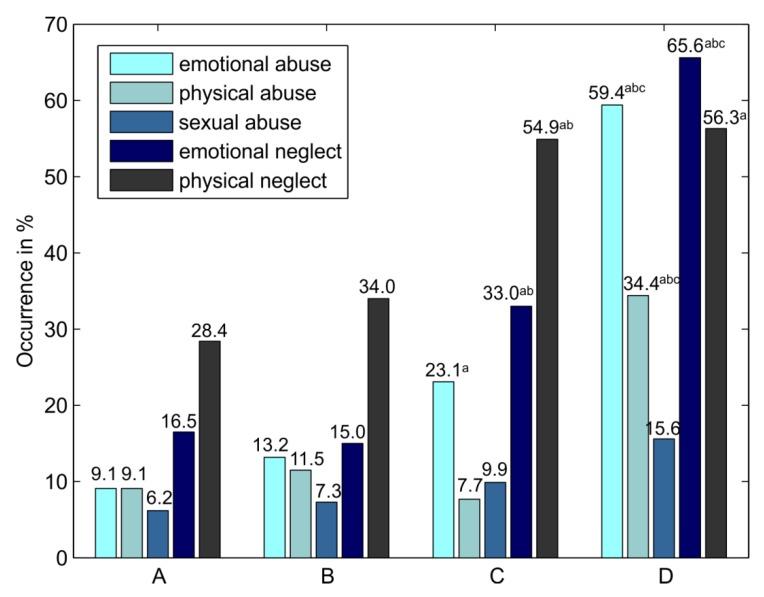
Prevalence of various childhood trauma types in the research groups. Note: Various types of childhood trauma as independent variables dichotomized according to Walker’s clinical cut-off scoring [48]. (**A**) Community sample reporting no chronic conditions (*n* = 405); (**B**) Community sample reporting other chronic conditions (*n* = 506); (**C**) Community sample reporting anxiety and pain (*n* = 91); (**D**) Clinical sample (respondents with a clinical diagnosis of anxiety or adjustment disorder who concurrently reported anxiety and some pain condition, *n* = 32). ^a^ *p* < 0.05 in comparison with group (**A**); ^b^ *p* < 0.05 in comparison with group (**B**); ^c^ *p* < 0.05 in comparison with group (**C**); Differences in occurrence of childhood trauma types between groups assessed by a test of proportions with Bonferroni correction.

The prevalence of various types of childhood trauma and differences between groups are depicted in Figure 2. The prevalence of reporting emotional abuse, emotional neglect and physical neglect was significantly higher in the community sample reporting anxiety and pain in comparison to the community sample reporting no chronic conditions. The prevalence of reporting emotional neglect and physical neglect was significantly higher in the community sample reporting anxiety and pain than in the community sample with other chronic conditions. The prevalence of reporting emotional and physical abuse and emotional and physical neglect was significantly higher in the clinical sample than in the community sample reporting no chronic conditions. The prevalence of emotional abuse, physical abuse, and emotional neglect was significantly higher in the clinical sample in comparison with all three community samples.

### 3.3. Odds of Reporting Anxiety and Pain in the Community and Clinical Samples in Subjects Reporting Various Types of Childhood Trauma

The odds of reporting anxiety and pain in the community and clinical samples in subjects reporting various types of childhood trauma are presented in Table 2.

Respondents reporting emotional abuse, emotional neglect, or physical neglect have higher odds of being in the community sample reporting anxiety and pain than in the community sample reporting no chronic conditions (group C vs. group A, OR/CI: 3.79/2.02–7.12; 2.42/1.41–4.14; 2.55/1.56–4.15) or in the community sample reporting chronic conditions other than anxiety or/and pain (group C vs. group B, OR/CI: 2.14/1.21–3.77; 2.78/1.67–4.63; 2.24/1.41–3.55).

Respondents reporting emotional abuse, physical abuse, emotional neglect or physical neglect have higher odds of being in the clinical sample reporting anxiety and pain than in the community sample reporting no chronic conditions (group D vs. group A; OR/CI: 14.71/6.56–32.95; 5.23/2.3–11.89; 9.56/4.35–21.00; 3.30/1.57–6.95) or in the community sample reporting chronic conditions other than anxiety or/and pain (group D vs. group B; OR/CI: 8.28/3.83–17.90; 4.04/1.82–8.96; 10.99/5.03–24.03; 2.9/1.39–6.06). For respondents reporting sexual abuse, the odds of being in the clinical group compared to the community sample with no chronic conditions has borderline significance (group D vs. group A; OR/CI 2.51/0.88–7.20; *p* = 0.087).

Respondents reporting emotional abuse, physical abuse, or emotional neglect have higher odds of being in the clinical sample reporting anxiety and pain than in the community sample reporting anxiety and pain (group D vs. group C; OR/CI: 3.88/1.61–9.31; 6.39/2.18–18.74; 3.95/1.67–9.36).

Moreover, respondents reporting emotional abuse have higher odds of being in the community sample reporting other chronic conditions than in the community sample with no chronic conditions (group B vs. group A; OR/CI 1.77/1.14–2.75, *p* = 0.011, not included in Table 2).

## 4. Discussion

In the current study, we investigated the relationship between history of childhood trauma and anxiety and long-term pain both in community and clinical populations. Reporting anxiety and pain was associated with a higher prevalence of emotional abuse and emotional and physical neglect in the community population and with a higher prevalence of emotional and physical abuse and emotional and physical neglect in the clinical population. Individuals from the general population reporting emotional abuse and emotional and physical neglect have higher odds of reporting anxiety and some chronic pain condition compared to individuals reporting no chronic conditions but also compared to those who have some other chronic condition but not anxiety or/and pain. Individuals reporting emotional and physical abuse and emotional neglect in particular have higher odds of suffering from an anxiety or adjustment disorder with concurrent long-term pain compared to the general population reporting anxiety and pain.

There is a lot of evidence from population studies on the higher occurrence of childhood trauma in people with chronic pain [1,2] and anxiety in adulthood [6,43]. In our study, there is higher occurrence of emotional abuse and both emotional and physical neglect in the population reporting anxiety and pain compared to healthy people and a higher occurrence of both types of neglect compared to the population with other chronic problems. When assessing the odds of reporting anxiety and chronic pain in the community sample by taking into account age and gender, reporting ‘emotional abuse’ and ‘emotional and physical neglect’ is associated with higher odds of reporting anxiety and chronic pain compared to the ‘healthy’ population but also compared to the population reporting other chronic conditions. This finding is supported by results from a meta-analytic review [1], in which individuals from the community reporting pain were more likely to have been abused or neglected than individuals from the community not reporting pain. In a prospective Canadian cohort study [50], persons reporting multiple stressful experiences in childhood were at increased risk of developing chronic back pain. A recent German population study [2] showed significant associations between all forms of childhood trauma and pain; however, the largest effect sizes were found for the correlation between emotional abuse and pain levels, with anxiety having an independent association with pain.

Anxiety in relation to pain can be associated with the tendency to catastrophize pain [29], which can contribute to pain chronification [29,30]. People who experienced early trauma may be prone to develop more anxious symptoms as a reaction to the experience of chronic pain. Furthermore, taking into account the bidirectional relationship between pain and anxiety [28] and possible shared risk factors, including childhood trauma [9,12], people with a history of childhood trauma may be vulnerable to developing both anxiety and pain symptoms later in life. Researchers in a survey study of adult internal medicine outpatients [44] found that emotional abuse in particular is relevant for self-reported pain and catastrophizing pain.

Studies based on clinical samples indicate a higher occurrence of childhood trauma in anxiety disorders and a significant relationship between childhood trauma and adulthood anxiety [6,33,51]. Even though in the current study the clinical sample with 32 patients was relatively small, we found a higher proportion of emotional abuse and neglect and physical abuse compared to healthy people and compared to the community reporting some chronic conditions with/without pain and anxiety. Reporting all types of abuse and neglect was associated with higher odds of suffering from a clinically diagnosed disorder with concurrent anxiety and pain compared to the healthy population and, with exception of sexual abuse, also compared to the community reporting other chronic conditions. When comparing with the community sample reporting anxiety and pain, only ‘emotional abuse and neglect’ and ‘physical abuse’ were associated with higher odds of suffering from a clinically diagnosed disorder. Our findings are in line with the growing evidence that emotional abuse and neglect have profound adverse effects on health [6,43]. ‘Emotional abuse’, including parental verbal abuse, has received little attention and seems to be a more elusive and insidious form of abuse than the more studied and visible physical and sexual abuse [52], but devaluing and hurtful words can have a profoundly negative impact on self-image and self-esteem. Many clinicians working with patients with anxiety symptoms notice their negative self-image and low self-esteem. ‘Emotional neglect’ is qualitatively different from abuse, because it is associated with a lack of appropriate stimulation and interaction. Neglecting of a child’s emotional needs in early infancy can alter the development of brain reward and oxytocin systems [53], which can have negative consequences on later health.

Surprisingly, there was no association between ‘physical abuse’ and reporting anxiety and pain in the community sample, which was in contrast with studies stating the relationship between physical abuse and pain in samples of women [41,54]. However, as expected, physical abuse was a strong predictor for suffering from a clinically diagnosed disorder, which was in line with the finding from a recent prospective study showing associations between physical abuse and anxiety disorders [6].

Similarly, there was no association between ‘sexual abuse’ and reporting anxiety and pain in the community sample, which was in line with a Canadian study investigating the association of sexual abuse to pain on a sample of women [54]. Sexual abuse was a weak predictor, with borderline significance for suffering from a clinical disorder, which is in line with findings from a prospective Australian study [6] and in contrast with an American National Comorbidity Survey [55], which found a strong relationship between sexual abuse and anxiety disorders. Because sexual abuse had the smallest percentages from all the types of childhood trauma in our study, it may cause the lower statistical power in our analyses. A possible explanation for the smaller occurrence of sexual abuse is that it is underreported due to secrecy and stigma. On the other hand, in many individual cases it may be better recognized at an earlier age before leading to long-term consequences.

Our findings of higher odds of suffering from an anxiety or adjustment disorder by reporting childhood trauma are supported by the findings from a systematic review [34], a Dutch study [33] and an American national study [32], which showed a strong association between childhood trauma and anxiety disorders. The strong relationship between anxiety disorders and chronic pain is well established from large representative and population-based studies [8,11,56]. In our study, respondents from the clinical population are people who have already sought professional help. Even though we do not have enough information about the respondents from the community (we do not know whether those reporting anxiety have been diagnosed with some clinical disorder accompanied by anxiety or depression), our findings indicate a stronger relationship of childhood trauma with anxiety and pain in the clinical sample. The concurrence of a genetic predisposition with psychosocial factors, namely childhood trauma [9], may be substantial in later development of a disorder with anxiety and long-term pain. Early adverse experiences are associated with some of the same biological alterations, including changes in neuroendocrine, neurotransmitter, and immunological systems, which influence anxiety disorders as well as pain-related conditions [36]. Thus, these common links between childhood trauma–anxiety and childhood trauma–chronic pain could be substantial in the relationship between trauma in childhood, anxiety in adulthood and long-term pain, the three items of the ‘unholy trinity’ we refer to.

Our results highlight the relevance of various types of childhood trauma as possible factors contributing to anxiety and pain symptoms in adulthood. Namely, emotional abuse and neglect and physical abuse were associated with anxiety and chronic pain in the clinical population. Greater awareness of these factors can be helpful in primary and secondary prevention in the area of public health as well as in treatment programs for people suffering from anxiety and chronic pain. Patients with anxiety and some chronic pain condition should be screened for the occurrence of childhood trauma, because it could be helpful in planning effective therapeutic strategies. In patients with chronic pain and a history of childhood trauma, multimodal therapeutic approaches comprised of—e.g., education about mechanisms maintaining chronic pain, better self-awareness training, fostering positive self-body image, relaxation techniques training, etc.—can be useful [57]. Primary prevention strategies centered on education of the general population regarding the negative health consequences of child abuse and neglect (especially less visible emotional abuse and neglect) and on increasing the responsiveness of the population to this problematic field could be valuable.

### Strengths and Limitations

The strength of this study is that it includes a comparison of a community sample reporting pain and anxiety with a clinical sample with diagnosed anxiety or adjustment disorder; furthermore, it analyzes all relevant types of childhood abuse and neglect in research samples.

The current study has several limitations. First, the status of a chronic pain condition was based on self-report of a diagnosis, which could be imprecise. On the other hand, self-reported checklists of chronic conditions have been commonly used in national studies, and the results suggest that the assessment of somatic diseases by self-reports is a valid option in mental–physical comorbidity research [58]. A second limitation is that a history of childhood trauma or life stressors was based on retrospective self-reports. Although there are controversial findings regarding potential recall and response bias, studies have shown a trend towards substantial under-reporting rather than over-reporting of childhood abuse and neglect in adulthood [59,60]. A third limitation is the fact that some self-reported chronic health conditions (e.g., cancer, gastric and duodenal ulcer, inflammatory bowel disease) in the control sample could be in individual cases related to long-lasting pain; nevertheless, they were not labeled as ‘pain-related’ in this study. Fourth, the clinical sample was relatively small and consisted of diagnoses of anxiety or adjustment disorders with concurrent anxiety and chronic pain. Participants with adjustment disorders may likely have a stronger relationship between symptoms and some later (not early) life stressors. Moreover, there could be more confounding factors (e.g., parent mental health, family violence, poverty) which were not assessed in this study.

## 5. Conclusions

Our results highlight the relevance of various types of childhood trauma as possible factors contributing to anxiety and pain-related conditions in adulthood. Reporting anxiety and pain was associated with a higher prevalence of emotional abuse and emotional and physical neglect in the community population and with a higher prevalence of emotional and physical abuse and emotional and physical neglect in the clinical population. Individuals reporting emotional and physical abuse and emotional neglect have higher odds of suffering from anxiety or adjustment disorder with concurrent long-term pain.

Further research should focus on a better understanding of the role of adulthood anxiety, attachment anxiety, depression, resilience, etc., as possible moderators and/or mediators between childhood trauma and chronic pain.

## Figures and Tables

**Table 1:** Sociodemographic characteristics of the community and clinical samples.

Sociodemographic Groups	(A) Community Sample Reporting No Chronic Conditions	(B) Community Sample Reporting Other Chronic Conditions	(C) Community Sample Reporting Anxiety and Pain *	(D) Clinical Sample ** Reporting Anxiety and Pain
	*n* = 405	*n* = 506	*n* = 91	*n* = 32
Age: Mean (SD)	36.4 (14.3)	46.3 (17.6)	51.6 (18.6)	39.1 (12.6)
Gender: *n* (%)				
1. Male	235 (58.0)	266 (52.6)	27 (29.7)	6 (18.8)
2. Female	170 (42.0)	240 (47.4)	64 (70.3)	26 (81.3)
Living arrangement: *n* (%)				
1. With a partner in marriage	152 (37.5)	266 (52.6)	39 (42.9)	11 (34.4)
2. With a partner	98 (24.2)	98 (19.4)	20 (22.0)	1 (3.1)
3. Alone	94 (23.2)	91 (18.0)	25 (27.5)	10 (31.3)
4. With parents, siblings	61 (15.1)	51 (10.1)	7 (7.7)	10 (31.3)
Education level: *n* (%)				
1. Primary	18 (4.4)	41 (8.1)	10 (11.0)	3 (9.4)
2. Skilled operative	81 (20.0)	100 (19.8)	33 (36.3)	9 (28.1)
3. High school, graduated	212 (52.3)	259 (51.2)	32 (35.2)	14 (43.8)
4. College/University	94 (23.2)	106 (20.9)	16 (17.6)	6 (18.8)

Note: SD = standard deviation; * community sample reporting anxiety and pain concurrently with possibly some other chronic condition; ** clinically diagnosed anxiety or adjustment disorder according to ICD-10.

**Table 2:** Odds of reporting anxiety and pain in the community and clinical samples in subjects reporting various types of childhood trauma (CTQ)

Childhood Trauma Questionnaire (CTQ)	Group (C) vs. (A)	Group (D) vs. (A)	Group (C) vs. (B)	Group (D) vs. (B)	Group (D) vs. (C)
	OR (95% CI)	OR (95% CI)	OR (95% CI)	OR (95% CI)	OR (95% CI)
Emotional abuse (cut-off score 10)	**3.79** (2.02‒7.12) ***	**14.71** (6.56–32.95) ***	**2.14** (1.21‒3.77) *	**8.28** (3.83‒17.90) ***	**3.88** (1.61‒9.31) **
Physical abuse (cut-off score 8)	0.82 (0.34‒1.95)	**5.23** (2.30‒11.89) ***	0.63 (0.28‒1.45)	**4.04** (1.82‒8.96) ***	**6.39** (2.18‒18.74) ***
Sexual abuse (cut-off score 8)	1.52 (0.69‒3.51)	2.51 (0.88‒7.20)	1.32 (0.61‒2.88)	2.19 (0.78‒6.13)	1.65 (0.50‒5.46)
Emotional neglect (cut-off score 15)	**2.42** (1.41‒4.14) ***	**9.56** (4.35‒21.00) ***	**2.78** (1.67‒4.63) ***	**10.99** (5.03‒24.03) ***	**3.95** (1.67‒9.36) **
Physical neglect (cut-off score 8)	**2.55** (1.56‒4.15) ***	**3.30** (1.57‒6.95) **	**2.24** (1.41‒3.55) ***	**2.90** (1.39‒6.06) **	1.30 (0.57‒2.96)

Note: Various types of childhood trauma as independent variables dichotomized according to Walker’s clinical cut-off scoring [48]. Multinomial logistic regression models were adjusted for gender and age. (A) Community sample reporting no chronic condition (*n* = 405); (B) Community sample reporting other chronic conditions (*n* = 506); (C) Community sample reporting concurrently anxiety and pain, (*n* = 91); (D) Clinical sample (respondents with a clinical diagnosis of anxiety or adjustment disorder who concurrently reported anxiety and some pain condition, *n* = 32). OR = odds ratio; 95% CI = 95% confidence interval of the odds ratio; * *p* < 0.05, ** *p* < 0.01, *** *p* < 0.001.
